# NUCKS1, a LINC00629-upregulated gene, facilitated osteosarcoma progression and metastasis by elevating asparagine synthesis

**DOI:** 10.1038/s41419-023-06010-9

**Published:** 2023-08-01

**Authors:** Shuo Zheng, Renchen Ji, Hongtao He, Na Li, Chuanchun Han, Jian Han, Xiaodong Li, Lu Zhang, Yuan Wang, Wenzhi Zhao

**Affiliations:** 1 The Second Affiliated Hospital, Dalian Medical University, Dalian, Liaoning 116044 P.R. China; 2 Institute of Cancer Stem Cell, Dalian Medical University, Dalian, Liaoning 116044 P.R. China; 3 National-Local Joint Engineering Research Center for Drug-Research and Development (R&D) of Neurodegenerative Diseases, Dalian Medical University, Dalian, 116044 People’s Republic of China; 4 Department of Orthopedics, The Third People’s Hospital of Dalian, Dalian Medical University, Dalian, Liaoning 116033 P.R. China

**Keywords:** Bone cancer, Cell growth

## Abstract

Nuclear ubiquitous casein and cyclin-dependent kinase substrate 1 (NUCKS1) has been reported to play an oncogenic role in several cancers. However, the biological functions and regulatory mechanism of NUCKS1 in osteosarcoma have not been fully understood. In this study, we reported that NUCKS1 was significantly increased in osteosarcoma. Depletion of NUCKS1 decreased osteosarcoma cell proliferation and metastasis in vivo and in vitro. Overexpression of NUCKS1 accelerated osteosarcoma cell aggressiveness. Mechanistically, NUCKS1 facilitated asparagine (Asn) synthesis by transcriptionally upregulating asparagine synthetase (ASNS) expression and elevating the levels of Asn in osteosarcoma cells, leading to increased cell growth and metastasis. Inhibition of ASNS or reduction of Asn decreased osteosarcoma cell aggressiveness and impaired the promoting effects of NUCKS1 on tumorigenesis and metastasis. Furthermore, we also found that by acting as a sponge for miR-4768-3p, LINC00629 promoted NUCKS1 expression. Collectively, our findings highlight the role of NUCKS1 in regulating asparagine metabolism and reveal that LINC00629 is an important regulator of NUCKS1 that contributes to NUCKS1 upregulation in osteosarcoma.

## Introduction

Osteosarcoma (OS) is the most common malignant bone tumor and primarily affects young, growing adolescents. However,it also has a second peak of incidence in people aged 50 or older with pre‐existing bone‐deforming conditions such as Paget’s disease [1, 2]. The 5-year relative survival rate of patients with localized osteosarcoma is ~60–70% due to surgery and multi-agent chemotherapy [3, 4]. However, after the development of metastases, the 5-year survival rate drops to less than 20%. Thus, uncovering the mechanism of metastasis and identifying new targets are essential for osteosarcoma therapy.

NUCKS1, located on human chromosome 1q32.1, belongs to the high mobility group family of proteins and is present in various vertebrate cell types and tissues [5, 6]. NUCKS1 is a phosphorylation substrate for cyclin-dependent kinases, casein kinase 2 (CDK2), DNA damage response (DDR) kinases ATM, and DNA-activated protein kinase (DNA-PK) [7, 8]. NUCKS1 plays a key role in the regulation of glucose metabolism and energy homeostasis, and the expression of NUCKS1 is inversely associated with body mass index in humans and body fat in mice. In addition, NUCKS1 is reported to act as a transcription factor that regulates insulin receptor signaling and the cell cycle [9–12]. Interestingly, NUCKS1 plays an oncogenic role in a number of cancers, including cervical squamous cell carcinoma [13], breast cancer [14, 15], lung cancer [16], colorectal cancer [17, 18], gastric cancer [19], and hepatocellular carcinoma [20, 21]. However, its functions and regulatory mechanism in osteosarcoma remain unclear.

Asparagine synthetase (ASNS) catalyzes the ATP-dependent conversion of aspartate and glutamine to asparagine and glutamate [22]. Clinically, ASNS dysfunction is associated with childhood acute lymphoblastic leukemia (ALL) and asparagine synthetase deficiency (ASD) [23–25]. ASNS and asparagine are also crucial for many solid tumors. For example, ASNS was increased and promoted cell metastasis by regulating β-catenin stability in lung cancer. Forced expression of ASNS reduced lung cancer cell apoptosis and cell cycle arrest under nutrition stress [26, 27]. Downregulation of ASNS suppressed cell proliferation in breast cancer and gastric cancer [28–30]. In colorectal cancer, SOX12 facilitates cell proliferation and metastasis by upregulating ASNS expression [31]. Although ASNS was reported to be essential for the progression of several tumors, its role and underlying mechanisms in osteosarcoma are still poorly understood.

This study reports that NUCKS1 is increased in osteosarcoma tissue and promotes asparagine synthesis via transcriptional upregulation of ASNS expression. High expression of NUCKS1 also promotes osteosarcoma cell survival and tumor formation. The study also indicates that LINC00629 upregulates NUCKS1 expression by “sponging” miR-4768-3p, leading to an increase in ASNS. Therefore, our results uncover a novel role of NUCKS1 in regulating asparagine metabolism and identify LINC00629 as an important regulator of NUCKS1 upregulation in osteosarcoma.

## Methods

### Cell culture and reagents

Human osteosarcoma cell 143B were maintained in Dulbecco’s modified Eagle medium (DMEM). MNNG/HOS cells were cultured in Minimum Essential Medium (MEM). These media were supplemented with 10% foetal bovine serum (FBS), 2 mM l-glutamine, penicillin (100 U/ml), and streptomycin (100 μg/ml) in a humidified atmosphere of 5% CO_2_ maintained at 37 °C. The following antibodies were used in this study: antibodies against GAPDH (Santa Cruz Biotechnology, Dallas, TX, USA; SC-25778, 1:1000), NUCKS1 (Proteintech, 12023-2-AP, 1:1000), ASNS (Proteintech, 14681-1-AP, 1:1000), Ki67 (Abcam, ab15580,1:500), Cleaved Caspase 3 (Cell Signaling, #9661, 1:400), l-Asparagine monohydrate (Sigma, A8381), l-Glutamate (Sigma, G1251), l-Asparaginase (MCE, HY-P1923).

### Lentivirus packaging and infection

To generate the lentiviral shRNA constructs against human LINC00629, NUCKS1, and ASNS, the target sequences were cloned into pLKO.1-puro vector. The shRNA sequences were listed: shRNA sequences for LINC00629 #1:5-CGTGAGTTTATAAGCGGAT-3, #2: 5-GGGTTGTAGTAGGTGTATA-3; shRNA sequences for ASNS #1: 5-GCTGTATGTTCAGAAGCTAAA-3, #2: 5-CGAGTGAAGAAATATCCGTAT-3; shRNA sequences for NUCKS1 #1:5-CATTTCTCTCTCTCTCTCTTT-3, #2: 5-GTTGTTGATTACTCACAGTTT-3. To generate the lentiviral expression vector for LINC00629 or NUCKS1, it was constructed into a pCDH vector. To establish a stable cell line, the pLKO.1 vector, pVSVG, pREV and pGAG or pCDH vector, psPax2, and pMD2G were co-transfected into HEK293T cells. Six hours after transfection, we changed the media to 8 ml DMEM/20% FBS per 10 cm plate and incubated additional 48 h to generate lentivirus. 48 h after post-media change, we harvested the viral supernatant and spun down at 1500 rpm for 10 mins and collected the supernatant. Then, we added the supernatant into the osteosarcoma cells as indicated. 24 h after infection, osteosarcoma cells were cultured in medium containing 2.5 mg/ml puromycin for the selection of stable clones. The knockdown or overexpression efficiency was evaluated. To establish the NUCKS1/shRNA ASNS cells, we generated the lentiviral shRNA constructs against human ASNS using FG12 vector. We added the viral supernatant containing FG12 shRNA ASNS into NUCKS1 overexpressed cells. The expression of ASNS and NUCKS1 were detected by Western Blotting.

### Quantitative reverse transcription PCR

Total RNA was isolated using Trizol (Invitrogen). One microgram of total RNA was used to synthesize cDNA using the PrimeScriptTM RT reagent kit (Takara, RR047A) according to the manufacturer’s instructions. The primers were as follows: ASNS Up: 5-AGGAGAGTGAGAGGCTTCTG-3; Dn: 5- GGTGGCAGAGACAAGTAATAGG-3. NUCKS1 Up: 5-TGCCCAAACCCAGACTAAAG-3, Dn:5-CCTTTGATGCCTTTGAAGCTG-3; Actin up:5- ATCAAGATCATTGCTCCTCCTGAG-3, Dn: 5-CTGCTTGCTGATCCACATCTG-3. The primers for miR-4768-3p were purchased from Takara. The expression levels of these genes were normalized to those of β-actin for genes, or U6 for miRNA. Changes in gene expression were determined using the 2^−ΔΔCT^ method.

### Colony formation and cell migration assay

For the colony formation assay, the indicated 143B and MNNG/HOS cells were seeded into a six-well plate (3000 cell per well) and cultured at 37 °C in a 5% CO_2_ incubator for 1 week. Growth media were replenished every 48 h during a 1-week period. Then, cell colonies were fixed with methanol for 15 mins. Following PBS washes, cells were stained with 0.1% crystal violet for 15 mins. Images of cell colonies were captured using the Bio-Rad ChemiDoc XRS+ system and quantified with the ImageJ programme.

Cell migration assay was conducted and 10,000 cells were seeded in a 24-well Transwell plate with 8-mm polyethylene terephthalate membrane filters (Corning, 3422). The cells resuspended in 200 μL serum-free media were seeded into the upper chamber, and a total of 650 μL complete medium supplemented with 10% FBS was added into the lower chamber. After incubation at 37 °C with 5% CO_2_, the cells that passed through the membrane were fixed with 4% formaldehyde for 30 mins and stained with 0.1% crystal violet for 20 mins. After wiping off the upper layer of non-migrated or non-invasive cells with a cotton swab, cells were counted by light microscopy.

### Chromatin immunoprecipitation assay

MNNG/HOS Cells were crosslinked with 1% formaldehyde for 10 mins at room temperature. The chromatin immunoprecipitation (ChIP) assay was performed according to the manufacturer’s instructions using the anti-NUCKS1 antibody and a kit (EZ-ChIP, 17-371 Millipore, Merck KGaA, Darmstadt Germany). Anti-rabbit IgG was used as the control. The bound DNA fragments were eluted and amplified by quantitative reverse transcription PCR.

### RNA sequencing analysis and label-free quantitative proteomics

MNNG/HOS cells with or without NUCKS1 knockdown were collected and transported to BioMaker. RNA extraction, library construction, sequencing, and data analysis were performed by BioMaker, Beijing, China.

For label-free quantitative proteomics, 10^6^ MNNG/HOS cells with or without LINC00629 knockdown were collected and transported to Jingjie PTM Biolab, Hangzhou, China.

### Promoter reporters and dual-luciferase assay

The promoter of ASNS and the matching mutant were constructed into a pGL3-basic vector. Luciferase activity was measured in a 1.5-ml Eppendorf tube with a Promega Dual-Luciferases Reporter Assay kit (Promega E1980) according to the manufacturer’s protocol after transfection. Relative Renilla luciferase activity was normalized to firefly luciferase activity.

To analyze NUCKS1 3’UTR activity, the NUCKS1 3’UTR were constructed into the pSICHECK2 vector. After that, 1 μg of total plasmids was transfected into osteosarcoma cells together with miR-4768-3p mimics or inhibitor. Following transfection for 24 h, luciferase activity was measured in a 1.5 ml Eppendorf tube using the Promega dual-luciferase reporter assay kit (Promega E1980) according to the manufacturer’s protocol.

### In vivo tumorigenesis and metastasis assays

Male nude mice (4–6 weeks old, 18–20 g) were obtained from the SPF Laboratory Animal Center of Dalian Medical University (Dalian, China) and were randomly divided into the indicated groups. The indicated osteosarcoma cells (1 × 10^6^ per mouse) resuspended in 100 μl Phosphate Buffer Saline (PBS) were subcutaneously injected into nude mice. After 10 or 13 days, the size of the tumor was measured by Vernier callipers every 2 or 3 days and converted to TV according to the following formula: TV (mm^3^) = (*a* × *b*^2^)/2, where *a* and *b* are the maximum and minimum diameters, respectively. All animals were euthanized 25 or 16 days after the injection, and the transplanted tumors were removed, weighed and divided into two for further study.

For the in vivo metastasis assay: The indicated osteosarcoma cells (1 × 10^6^ per mouse) resuspended in 100 μl PBS were injected into nude mice through the lateral tail vein. All animals were euthanized 30 days after the injection. The lungs were fixed with 4% formalin and embedded in paraffin blocks. The metastatic lesions were confirmed by histological analysis. For animal experiments, all animal procedures were performed in accordance with the institutional guidelines for the care and use of laboratory animals approved by the Animal Care and Use Committee of Dalian Medical University.

### Bioinformatics analysis

In this study, we used the TNMplot (https://tnmplot.com) database to analyze the RNA expression of NUCKS1 and ASNS in normal(*n* = 564) and osteosarcoma(*n* = 88) tissues. We used the Kaplan–Meier plotter (KM plotter, http://kmplot.com) database or GEPIA database (gene expression profiling interactive analysis, http://cancer-pku.cn) to analyze the relationship between NUCKS1 or ASNS expression and prognoses in the patient with sarcoma.

### In vitro measurement of ASNS enzyme activity

Osteosarcoma cells as indicated were collected in 1.5 ml tubes and 1/15 of them were prepared for protein quantification by BCA assay, and the rest were washed three times with PBS and placed on ice. Each sample was resuspended in 400 μl sample buffer (50 mM Tris HCl, pH 8.0, 0.5 mM EDTA, 1 mM EGTA, 1 mM DTT and 1 mM PMSF), frozen in liquid nitrogen and dissolved in room temperature for four times. Cell extracts were centrifuged at 12,000 × g for 30 mins at 4 °C, and the supernatants were applied to analyze the enzymatic activity of ASNS by reacting with a substrate mixture (85 mM Tris HCl, pH 8.0, 50 mM NaCl, 8.33 mM MgC12, 5 mM ATP, 10 mM aspartic acid and 5 mM glutamine). In each reaction, 40 μl of enzyme solution were added to 20 μl substrate mixture and incubated with slight oscillation at 37 °C for 60 mins. Each reaction was done in triplicate. The reaction system was placed on ice and each was mixed with three times the volume of acetonitrile, followed by centrifuging twice at 14,000×*g* for 10 mins to extract metabolites. Asn and Glu were identified and quantified by a Triple Quadrupole LC/MS System according to calibration curve. ASNS activity was obtained by calculating the production of Asn by cell extracts with determined amount of protein in certain period. And the unit for ASNS activity is μg of Asn per minute per μg total protein (μg min/1 μg per protein).

### Ethynyldeoxyuridine assay

The effects of Asn and Glu on cell proliferation in MNNG/HOS cells with or without NUCKS1 knockdown were assessed using ethynyldeoxyuridine (EdU) detection kit (RiboBio, Guangzhou, China). The EdU incorporation rate was calculated as the ratio of the number of EdU‐incorporated cells to the number of Hoechst 33342‐staining cells.

### Immunohistochemistry

Human osteosarcoma and bone tissue microarrays (TMAs) were purchased from bioaitech (Xi’an, China; Catalogue Number: L714901 and LN020Bn01). These TMAs (L714901) contained 70 osteosarcoma tissues and 1 bone tissue. LN020Bn01 contained 10 bone tissues. We used the NUCKS1 and ASNS antibodies to perform immunohistochemical (IHC) staining on the same paraffin-embedded tissue blocks that were used for clinical diagnosis. Immunohistochemistry was performed using the avidin–biotin complex method (Vector Laboratories), including heat-induced antigen retrieval procedures.

### Statistics and data analyses

The data were expressed as the mean ± SD, and were statistically evaluated using GraphPad Prism 5. Multiple comparisons between treatment groups and control groups were performed using Dunnett’s least significant difference (LSD) test. Values of *p* < 0.05 were considered statistically significant.

## Results

### NUCKS1 promotes osteosarcoma cell growth and metastasis in vitro and in vivo

**Fig. 1:**
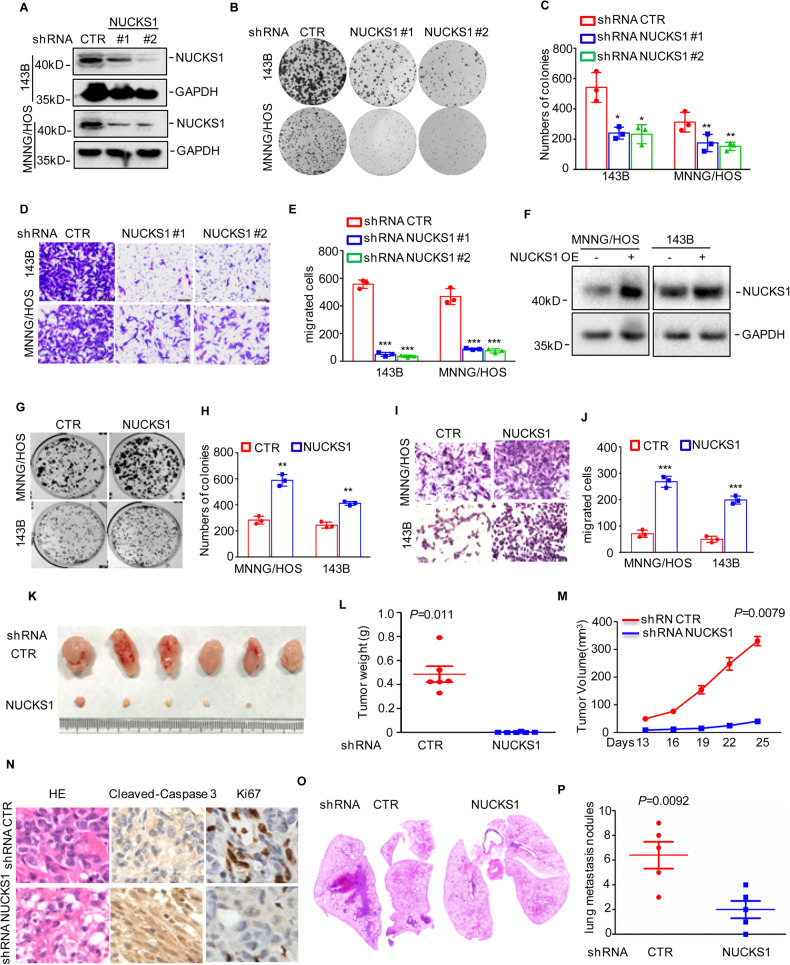
NUCKS1 promoted osteosarcoma cell growth and migration in vitro and in vivo. **A** NUCKS1 was knocked down in 143B and MNNG/HOS cells. The expression levels of NUCKS1 were detected by western blot. **B**, **C** 143B and MNNG/HOS cells with or without NUCKS1 depletion were tested for cell growth in the colony formation assay. After 1 week, viable colonies were counted and are shown (**B**). Data are depicted as bar graphs (**C**). **D**, **E** The migration of the indicated cells was detected by Transwell assays. Representative images of crystal violet-stained culture plates are shown (**D**). Data are depicted as bar graphs (**E**). **F** NUCKS1 was overexpressed in 143B and MNNG/HOS cells. The expression levels of NUCKS1 were detected by Western blot. **G**, **H** 143B and MNNG/HOS cells with or without NUCKS1 overexpression were tested for cell growth in the colony formation assay. After 1 week, viable colonies were counted and are shown (**G**). Data are depicted as bar graphs (**H**). **I**, **J** The migration of the indicated cells was detected by Transwell assays. Representative images of crystal violet-stained culture plates are shown (**I**). Data are depicted as bar graphs (**J**). **K**–**M** 143B cells (10^6^ cells per mouse) with or without NUCKS1 knockdown were injected subcutaneously into nude mice (*n* = 6). Representative images of xenograft tumors (**K**). The weight (**L**) and volume (**M**) of the tumors were calculated and analyzed. **N** Tumor tissue sections were analyzed by IHC using anti-Ki-67 and anti-Cleaved Caspase-3 antibodies. **O**, **P** 143B cells (10^6^ cells per mouse) with or without NUCKS1 knockdown were injected intravenously into nude mice (*n* = 5 per group). HE staining are displayed (**O**). Each group of metastatic nodules was assessed (**P**). Data in **C**, **E**, **H**, **J**, **L**, **M** and **P** were analyzed by Student’s *t* test, *p < 0.05, **p < 0.01, ***p < 0.001.

To evaluate the contribution of NUCKS1 to the development of human osteosarcoma, we first knocked down NUCKS1 with two independent shRNAs in 143B and MNNG/HOS cells. Compared with the control group, the expression of NUCKS1 in the shRNA groups was substantially reduced (Fig. 1A). Then, colony formation assays were performed to investigate the effects of NUCKS1 on cell growth. As shown in Fig. 1B, C, inhibition of NUCKS1 expression significantly decreased the number of colonies compared with the control group. In subsequent Transwell assays, the extent of migration of NUCKS1-depleted cells was remarkably lower than that of control cells (Fig. 1D, E). To further confirm this hypothesis, we overexpressed NUCKS1 in 143B and MNNG/HOS cells and verified the overexpression of NUCKS1 by western blotting (Fig. 1F). Consistent with an oncogenic role of NUCKS1 in osteosarcoma cells, elevated NUCKS1 expression promoted cell proliferation and migration, as indicated by an increase in the number of colonies and migrated cells (Fig. 1G–J).

To better understand the role of NUCKS1 in osteosarcoma, we subsequently investigated the effects of NUCKS1 on tumorigenesis and metastasis in vivo. We first implanted 143B cells with or without NUCKS1 knockdown into nude mice. Compared with control cells, 143B cells with NUCKS1 knockdown significantly suppressed tumor formation, as indicated by reduced tumor weights and sizes (Fig. 1K–M). The subsequent immunohistochemistry analyses assay was performed to detect the resected 143B xenograft tumor tissues from the control and NUCKS1 knockdown groups using the Ki-67 and Cleaved Caspase 3 antibodies. As shown in Fig. 1N, NUCKS1 knockdown reduced the percentages of Ki-67-positive cells and increased the proportions of cleaved caspase 3-positive cells.

Then, we assessed the effects of NUCKS1 on lung metastasis using xenograft models. The 143B cells with or without NUCKS1 depletion were injected intravenously into nude mice. Similarly, NUCKS1-depleted cells exhibited significantly decreased lung metastasis abilities at approximately three weeks, as indicated by a notable decrease in the number of metastatic lesions and the average surface areas produced by NUCKS1-depleted cells (Fig. 1O–P). Collectively, these data indicate that NUCKS1 accelerated osteosarcoma cell growth and metastasis in vitro and in vivo.

### NUCKS1 is increased in human osteosarcoma

**Fig. 2:**
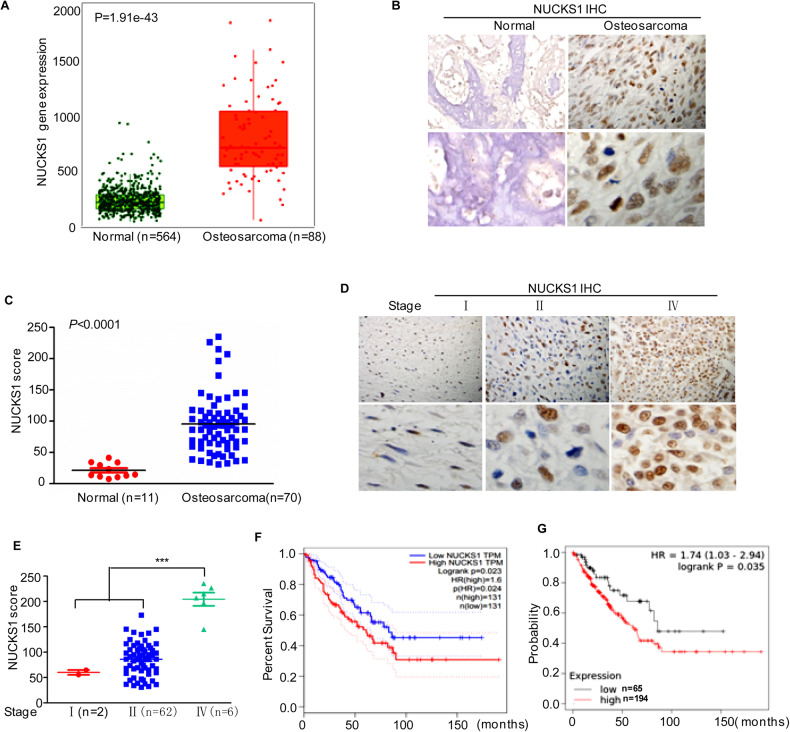
NUCKS1 is increased in osteosarcoma tissue. **A** The expression levels of NUCKS1 in osteosarcoma (*n* = 88) and normal tissues (*n* = 564) were analyzed from the TNMplot database. **B**, **C** Representative microphotographs of NUCKS1 immunohistochemical staining of normal bone tissues and osteosarcoma tissue sections (**B**). The expression of NUCKS1 normal bone tissues and osteosarcoma tissue was assessed (**C**). **D**, **E** Representative microphotographs of NUCKS1 immunohistochemical staining were performed in the indicated osteosarcoma tissues (**D**). The relationship between NUCKS1 expression and tumor staging was analyzed (**E**). **F** Kaplan–Meier plot of the overall survival rate of 262 patients with sarcoma. The data were obtained from the GEPIA database. **G** Kaplan–Meier plot of the overall survival rate of 259 patients with sarcoma. The data were obtained from the Kaplan–Meier Plotter. Data in **C** and **E** were analyzed by Student’s *t* test, *p < 0.05, **p < 0.01, ***p < 0.001.

To further confirm the oncogenic role of NUCKS1 in osteosarcoma, we first investigated NUCKS1 expression in the TNMplot database. As shown in Fig. 2A, compared with normal tissues, the expression of NUCKS1 was significantly increased in osteosarcoma. Then, we examined NUCKS1 expression in 70 formalin-fixed and paraffin-embedded osteosarcoma tissues and 11 normal bone tissues using immunohistochemical (IHC) staining. We found that NUCKS1 expression was higher than that in normal bone tissues (Fig. 2B, C). Furthermore, we investigated the relationship between clinicopathologic variables and NUCKS1 expression. The IHC results indicated that NUCKS1 expression was positively associated with the stage of osteosarcoma (Fig. 2D, E). In addition, we also found that sarcoma patients with relatively high NUCKS1 levels showed lower survival rates than patients with low NUCKS1 levels in the tumors (Fig. 2F, G). Taken together, these data strongly indicate that NUCKS1 is a key oncogene in osteosarcoma.

### NUCKS1 transcriptionally upregulated ASNS expression in osteosarcoma

**Fig. 3:**
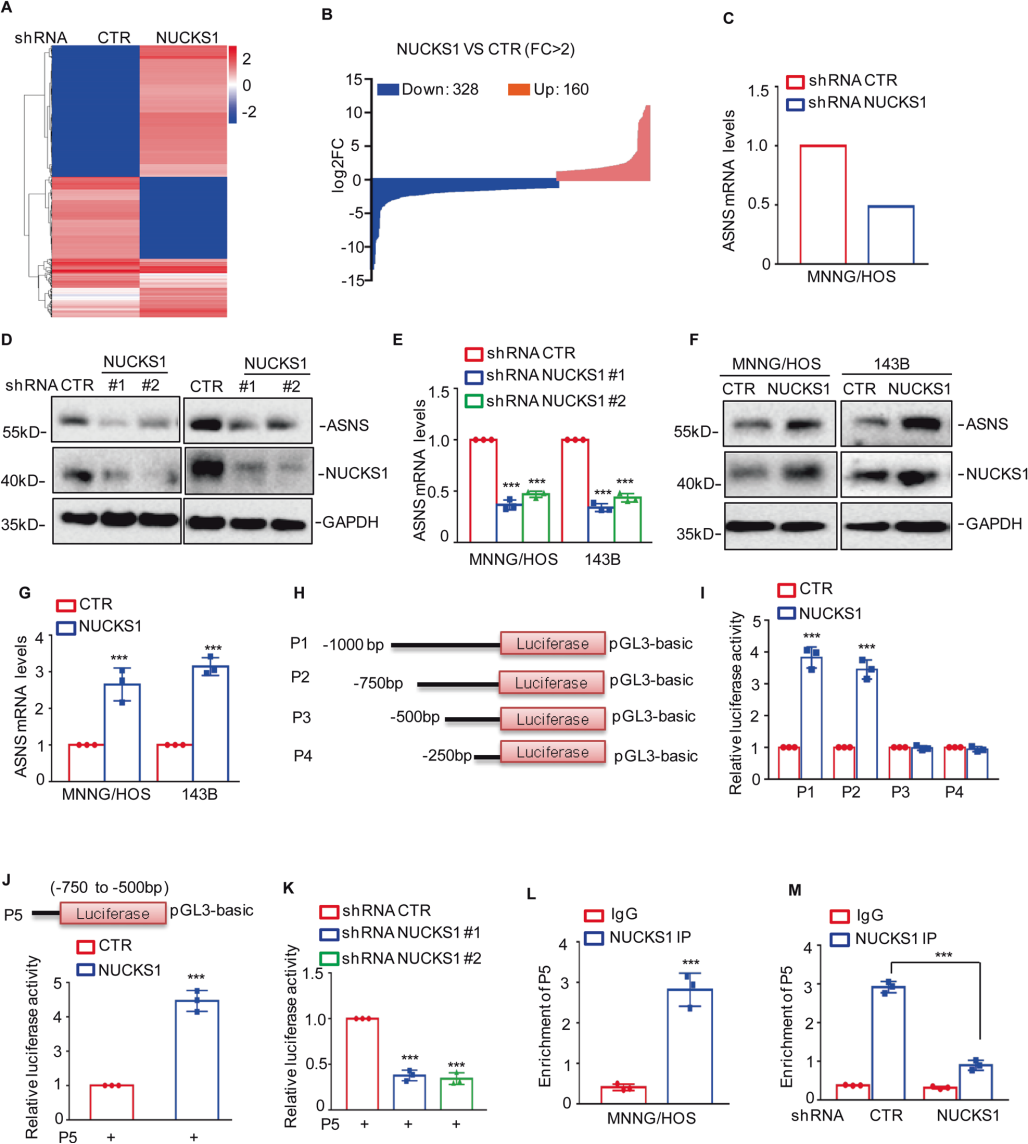
NUCKS1 transcriptionally upregulated ASNS expression. **A** Heatmap showing the altered genes identified by RNA sequencing analysis in NUCKS1-depleting MNNG/HOS cells. **B** The differentially expressed mRNAs for NUCKS1 knockdown versus the control were shown (absolute log2-fold change >1 and *q* value < 0.05). The blue indicates the downregulated genes, and the pink indicates the upregulated genes. **C** The mRNA levels of ASNS are shown from RNA sequencing data. **D**, **E** The protein and mRNA levels of ASNS were detected by western blot (**D**) and qRT‒PCR (**E**) in osteosarcoma cells with or without NUCKS1 knockdown. **F**, **G** The protein and mRNA levels of ASNS were detected by western blot (**F**) and qRT‒PCR (**G**) in osteosarcoma cells with or without NUCKS1 overexpression. **H** Schematic illustration of pGL3-based reporter constructs used in luciferase assays to examine the transcriptional activity of ASNS named P1, P2, P3, and P4. **I** P1, P2, P3, and P4 together with the *Renilla* luciferase plasmid were transfected into MNNG/HOS cells with or without NUCKS1 overexpression. The *Renilla* luciferase construct was used to control for transfection efficiency, and dual luciferase activity was measured. **J** Schematic illustration of pGL3-based reporter constructs used in luciferase assays to examine the transcriptional activity of ASNS named P5 (Up). P5 together with the *Renilla* luciferase plasmid was transfected into MNNG/HOS cells with or without NUCKS1 overexpression. Dual luciferase activity was measured. **K** P5 together with the *Renilla* luciferase plasmid was transfected into MNNG/HOS with or without NUCKS1 knockdown, and the cells were collected. Dual luciferase activity was measured. **L**, **M** ChIP analysis showed the binding of NUCKS1 to the promoter of ASNS in MNNG/HOS cells with or without NUCKS1 knockdown. Isotype-matched IgG was used as a negative control. Data in **E**, **G**, **I**–**M** were analyzed by Student’s *t* test, *p < 0.05, **p < 0.01, ***p < 0.001.

To dissect the underlying molecular mechanism whereby NUCKS1 promoted osteosarcoma cell tumorigenesis and metastasis, we used RNA-seq to identify the downstream genes of NUCKS1. After NUCKS1 knockdown, we obtained 328 downregulated and 160 upregulated genes (Fig. 3A, B and Supplementary Table 1). Among these altered genes, we found that ASNS was downregulated in NUCKS1-depleted cells (Fig. 3C). To confirm this hypothesis, we further detected ASNS expression in MNNG/HOS and 143B cells with or without NUCKS1 knockdown. Similarly, depletion of NUCKS1 observably downregulated the mRNA and protein levels of ASNS (Fig. 3D, E). In contrast, overexpression of NUCKS1 promoted ASNS expression (Fig. 3F, G).

To further validate that NUCKS1 transcriptionally upregulated ASNS expression, we inserted the upstream sequence of ASNS and different truncations into pGL3-based luciferase reporter plasmids (named P1, P2, P3, and P4) (Fig. 3H). These plasmids were then transfected into MNNG/HOS cells with or without NUCKS1 overexpression, and the luciferase activity of the ASNS promoter was measured. As shown in Fig. 3I, overexpression of NUCKS1 elevated the luciferase activities of P1 and P2 but not P3 and P4, suggesting that the region (−750 to −500 bp) was essential for NUCKS1-upregulated ASNS expression.

To confirm this hypothesis, we cloned the region (−750 to −500 bp) and inserted it into pGL3-based luciferase reporter plasmids, which were named P5. Subsequently, we transfected P5 into MNNG/HOS cells with or without NUCKS1 overexpression. As expected, increased NUCKS1 elevated the luciferase activity of P5 (Fig. 3J). However, depletion of NUCKS1 reduced the luciferase activity of P5 (Fig. 3K).

Furthermore, ChIP assays showed that the chromatin fragment containing p5 was specifically enriched in anti-NUCKS1 immunoprecipitation and that the binding capacity of NUCKS1 to the ASNS promoter was reduced in NUCKS1-depleted MNNG/HOS cells (Fig. 3L, M). Taken together, these findings indicate that NUCKS1 could bind the promoter of ASNS and upregulate ASNS expression in osteosarcoma cells.

### NUCKS1 elevates asparagine synthesis and alters osteosarcoma cell sensitivity to L-asparaginase

**Fig. 4:**
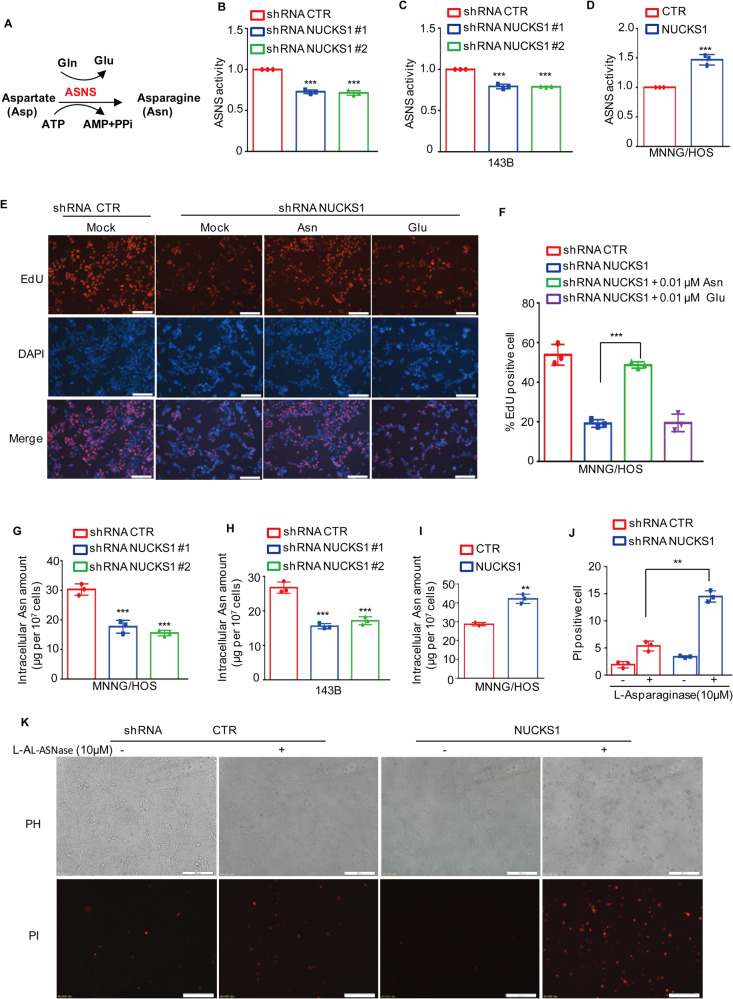
NUCKS1 elevates asparagine biosynthesis and alters sensitivity to L-asparaginase via ASNS. **A** Schematic diagram describing the reaction catalyzed by ASNS. **B**, **C** The relative enzyme activity of ASNS was assessed in MNNG/HOS and 143B cells with or without NUCKS1 knockdown. **D** The relative enzyme activity of ASNS was assessed in MNNG/HOS cells with or without NUCKS1 overexpression. **E**, **F** 0.01 mM aspartate (Asn) or glutamate (Glu) was added into NUCKS1-depleted cells. The proliferative abilities were measured with an EdU staining assay. Scale bar: 100 μm. **G**, **H** Intracellular amount of Asn was assessed in MNNG/HOS and 143B cells with or without NUCKS1 knockdown. **I** Intracellular amount of Asn was assessed in MNNG/HOS cells with or without NUCKS1 overexpression. **J**, **K** MNNG/HOS cells with or without NUCKS1 depletion were treated with 10 μM l-Asparaginase for 48 h. Cell death was measured by PI staining. Scale bar: 200 μm. Data in **B**–**D**, **F**–**J** were analyzed by Student’s *t* test, *p < 0.05, **p < 0.01, ***p < 0.001.

ASNS is a key enzyme that catalyzes the synthesis of asparagine (Asn) and glutamate (Glu) from aspartate (Asp) and glutamine (Gln) in an ATP-dependent reaction (Fig. 4A). We thus assessed the effects of NUCKS1 on the enzyme activities of ASNS in osteosarcoma cells. As shown in Fig. 4B, C, depletion of NUCKS1 reduced the enzyme activities of ASNS. Conversely, overexpression of NUCKS1 elevated ASNS activity (Fig. 4D). Then, we investigated whether the effects of NUCKS1 on cell proliferation were dependent on the production of ASNS. As shown in Fig. 4E, F, asparagine, but not glutamate, rescued the decrease in cell proliferation induced by NUCKS1 knockdown, suggesting that NUCKS1 promoted osteosarcoma cell growth in a manner dependent on asparagine synthesis. To confirm this hypothesis, we then evaluated the alteration of asparagine levels in NUCKS1-depleted or NUCKS1-overexpressing cells. Compared with the control cells, knockdown of NUCKS1 reduced the amounts of asparagine (Fig. 4G, H). However, forced NUCKS1 elevated the amounts of asparagine (Fig. 4I).

L-Asparaginase (L-ASNase) is an enzyme that hydrolyses asparagine into aspartic acid and ammonia, which leads to a reduction in asparagine. l-ASNase is reported to facilitate cell apoptosis in ASNS-deficient or ASNS-low tumor cells. Therefore, we investigated the alteration of sensitivity to l-ASNase in NUCKS1-depleted cells. Knockdown of NUCKS1 promoted l-ASNase-induced cell death (Fig.4J, K). Taken together, these findings indicate that NUCKS1 elevates asparagine synthesis and suppresses l-ASNase-induced osteosarcoma cell death.

### NUCKS1 promotes osteosarcoma cell tumorigenesis and metastasis partly dependent on upregulating ASNS expression

**Fig. 5:**
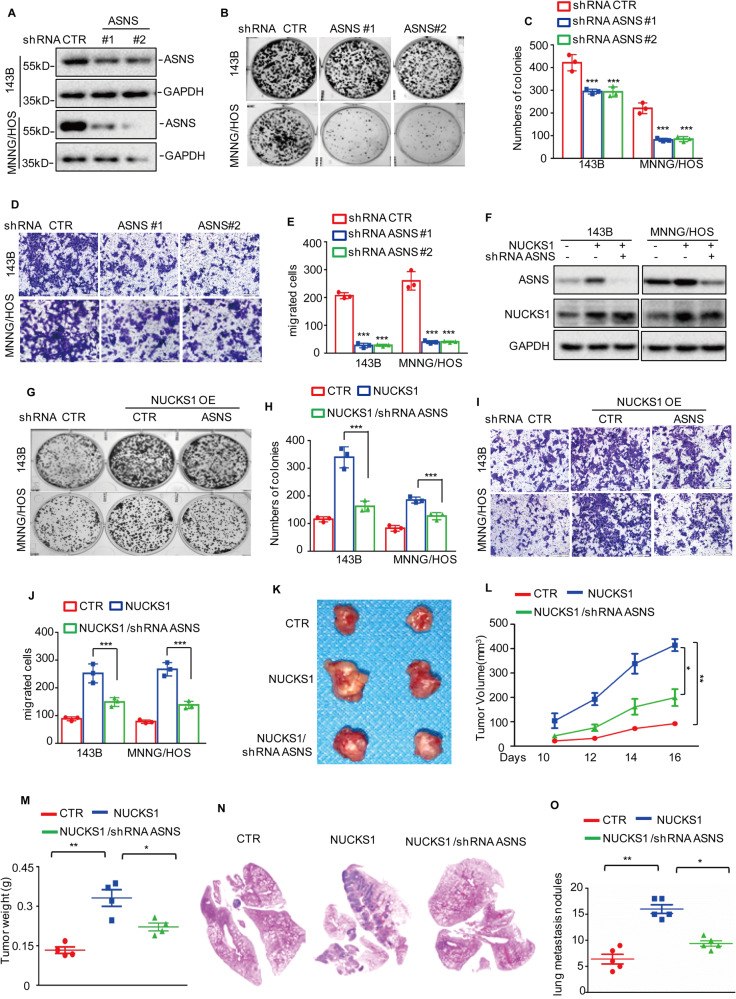
NUCKS1 promotes osteosarcoma tumorigenesis and metastasis via elevating ASNS expression. **A** ASNS was knocked down in 143B and MNNG/HOS cells. The expression levels of ASNS were detected by western blot. **B**, **C** 143B and MNNG/HOS cells with or without ASNS depletion were tested for cell growth in the colony formation assay. After 1 week, viable colonies were counted and are shown (**B**). Data are depicted as bar graphs (**C**). **D**, **E** The migration of the indicated cells was detected by Transwell assays. Representative images of crystal violet-stained culture plates are shown (**D**). Data are depicted as bar graphs (**E**). **F** ASNS was knocked down in 143B and MNNG/HOS cells with or without NUCKS1 overexpression. The expression levels of ASNS and NUCKS1 were detected by western blot. **G**, **H** Cell growth were assessed by the colony formation assay. **I**, **J** Cell migration was assessed by Transwell assay. **K**–**M** The indicated 143B cells (10^6^ cells per mouse) were injected subcutaneously into nude mice (*n* = 4). Representative images of xenograft tumors (**K**). The volume (**L**) and weight (**M**) of the tumors were calculated and analyzed. **N**–**O** The indicated143B cells (10^6^ cells per mouse) were injected intravenously into nude mice (*n* = 5 per group). HE staining are displayed (**N**). Each group of metastatic nodules was assessed (**O**). Data in **C**, **E**, **H**, **J**, **L**, **M**, and **O** were analyzed by Student’s *t* test, *p < 0.05, **p < 0.01, ***p < 0.001.

To determine whether NUCKS1 promotes osteosarcoma cell tumorigenesis and metastasis by upregulating ASNS, we first investigated the role of ASNS in osteosarcoma and knocked down ASNS with two independent shRNAs in 143B and MNNG/HOS cells. Compared with that in the control group, the expression of ASNS in the shRNA groups was substantially reduced (Fig. 5A). Then, we found that knockdown of ASNS suppressed osteosarcoma cell growth and migration (Fig. 5B–E). Subsequently, we overexpressed NUCKS1 in osteosarcoma cells with or without ASNS knockdown (Fig. 5F). Cell proliferation and migration were assessed by colony formation and Transwell assays. As shown in Fig. 5G–J, we found that inhibition of ASNS weakened the effects of NUCKS1 on cell growth and migration. Consistently, the reduction in Asn by l-ASNase also abolished the effects of NUCKS1 on cell proliferation and migration (Supplementary Fig. 1A–D).

Similar findings were observed in the xenograft tumor assay. Our findings indicated that the depletion of ASNS impaired the effects of NUCKS1 on tumor weight and volume (Fig. 5K–M). Likewise, the promotion of NUCKS1 on lung metastasis were impaired by ASNS depletion in nude mice (Fig. 5N–O and Supplementary Fig. 1E, F). Collectively, these results suggest that NUCKS1 promotes osteosarcoma cell growth and metastasis partly in a manner dependent on ASNS.

### Increased ASNS is positively associated with NUCKS1 expression in osteosarcoma and predicts a poor prognosis in sarcoma patients

**Fig. 6:**
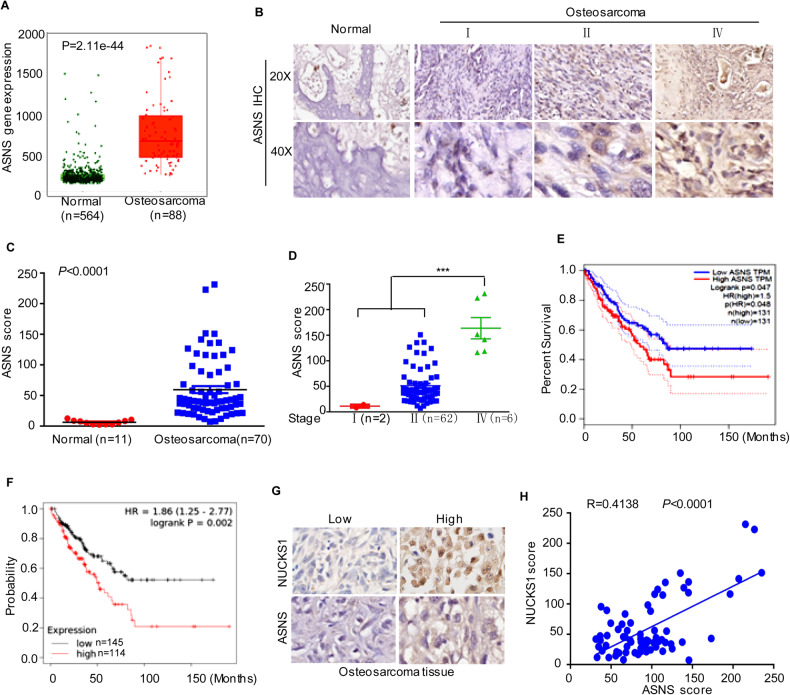
Increased ASNS is positively associated with NUCKS1 expression in osteosarcoma. **A** The expression levels of ASNS in osteosarcoma (*n* = 88) and normal tissues (*n* = 564) were analyzed from the TNMplot database. **B**, **C** Representative microphotographs of ASNS immunohistochemical staining of normal bone tissues and osteosarcoma tissue sections (**B**). The expression of ASNS in normal bone tissues and osteosarcoma tissue was assessed (**C**). **D** The relationship between ASNS expression and tumor staging was analyzed. **E** Kaplan–Meier plot of the overall survival rate of 262 patients with sarcoma. The data were obtained from the GEPIA database. **F** Kaplan–Meier plot of the overall survival rate of 259 patients with sarcoma. The data were obtained from the Kaplan–Meier Plotter. **G**, **H** Representative microphotographs of ASNS immunohistochemical staining of osteosarcoma tissues and the correction between ASNS and NUCKS1 were analyzed. Data in **C**, **D** and **H** were analyzed by Student’s *t* test, *p < 0.05, **p < 0.01, ***p < 0.001.

To evaluate the clinical importance of the NUCKS1-ASNS axis and determine their correlation in osteosarcoma, we first investigated ASNS expression in the TNMplot database. As shown in Fig. 6A, the expression levels of ASNS were significantly increased in osteosarcoma compared with normal tissues. Similarly, the IHC results indicated that ASNS expression in osteosarcoma tissues was higher than that in normal bone tissues (Fig. 6B, C). In particular, the expression levels of ASNS were positively associated with the staging of osteosarcoma (Fig. 6D).

To extend our findings, we investigated the association between ASNS expression and the overall survival of sarcoma patients. We found that patients with relatively high ASNS levels showed lower survival rates than patients with low ASNS levels (Fig. 6E, F). Furthermore, we explored the relationship between ASNS and NUCKS1 in osteosarcoma tissues. Our findings suggested that ASNS was upregulated in osteosarcoma tissues with high NUCKS1 expression compared with those with low NUCKS1 expression (Fig. 6G, H). Taken together, these data strongly verify that ASNS is increased in osteosarcoma tissues and that the expression of ASNS was positively associated with NUCKS1.

### LINC00629 upregulates NUCKS1 expression in osteosarcoma cells

**Fig. 7:**
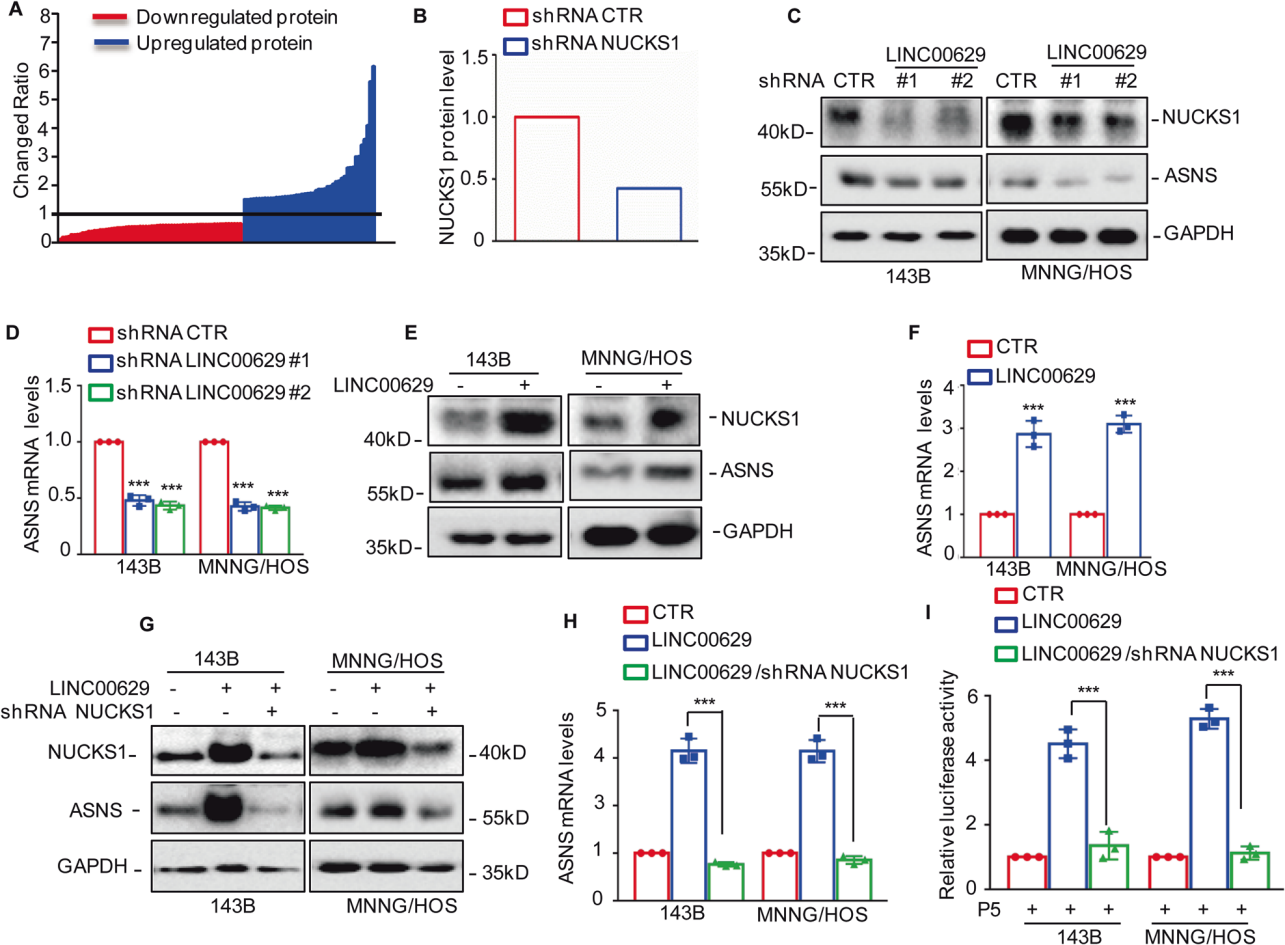
LINC00629 promotes NUCKS1 and ASNS expression in osteosarcoma cell. **A** The differentially expressed proteins in MNNG/HOS cells with or without LINC00629 knockdown were identified by label-free quantitative proteomics. The red line represented the downregulated genes and the blue line represented the upregulated genes. **B** The fold change in NUCKS1 was listed. **C**, **D** The expression levels of NUCKS1 and ASNS were detected by western blot and qRT-PCR in MNNG/HOS and 143B cells with or without LINC00629 knockdown. **E**, **F** The expression levels of NUCKS1 and ASNS were detected by western blot and qRT-PCR in MNNG/HOS and 143B cells with or without LINC00629 overexpression. **G**, **H** NUCKS1 was knocked down in MNNG/HOS and 143B cells with or without LINC00629 overexpression. The expression levels of NUCKS1 and ASNS were analyzed by western blot and qRT-PCR. **I** The promoter (−750 to −500bp) of ASNS(P5) was transfected into osteosarcoma cells as indicated. Dual luciferase activity was measured. Data in **D**, **F**, **H** and **I** were analyzed by Student’s *t* test, *p < 0.05, **p < 0.01, ***p < 0.001.

Recently, we uncovered the role of LINC00629 in osteosarcoma and identified differentially expressed proteins in MNNG/HOS cells with or without LINC00629 knockdown using label-free quantitative proteomics [32]. Among these altered proteins, we found that NUCKS1 was downregulated in LINC00629-depleted cells (Fig. 7A, B). To confirm it, we first detected NUCKS1 expression in LINC00629-depleted osteosarcoma cells. Compared with the control group, knockdown of LINC00629 reduced NUCKS1 and ASNS expression (Fig. 7C, D). Conversely, overexpression of LINC00629 facilitated NUCKS1 and ASNS expression (Fig. 7E, F).

To further investigate whether LINC00629-upregulated ASNS expression via NUCKS1, we then knocked down NUCKS1 expression in osteosarcoma cells with or without LINC00629 overexpression. The expression of ASNS was detected by Western blotting and qRT‒PCR. The results indicated that knockdown of NUCKS1 abolished the upregulation of ASNS by LINC00629 (Fig. 7G, H). Similarly, inhibition of NUCKS1 reversed the effects of LINC00629 on the promoter activity of ASNS (Fig. 7I). Collectively, these results revel that LINC00629 promotes NUCKS1 and ASNS expression in osteosarcoma cells.

### LINC00629 upregulates NUCKS1 by “sponging” miR-4768-3p

To uncover the molecular mechanism whereby LINC00629 upregulated NUCKS1, we first analyzed the mRNA levels of NUCKS1 in osteosarcoma cells with or without LINC00629 knockdown. As shown in Supplementary Fig. 2A, we found that depletion of LINC00629 had no effect on NUCKS1 mRNA expression. Then, we also investigated NUCKS1 stability in LINC00629-depleted cells and found that decreased LINC00629 had no effect on the stability of NUCKS1 (Supplementary Fig. 2B).

**Fig. 8:**
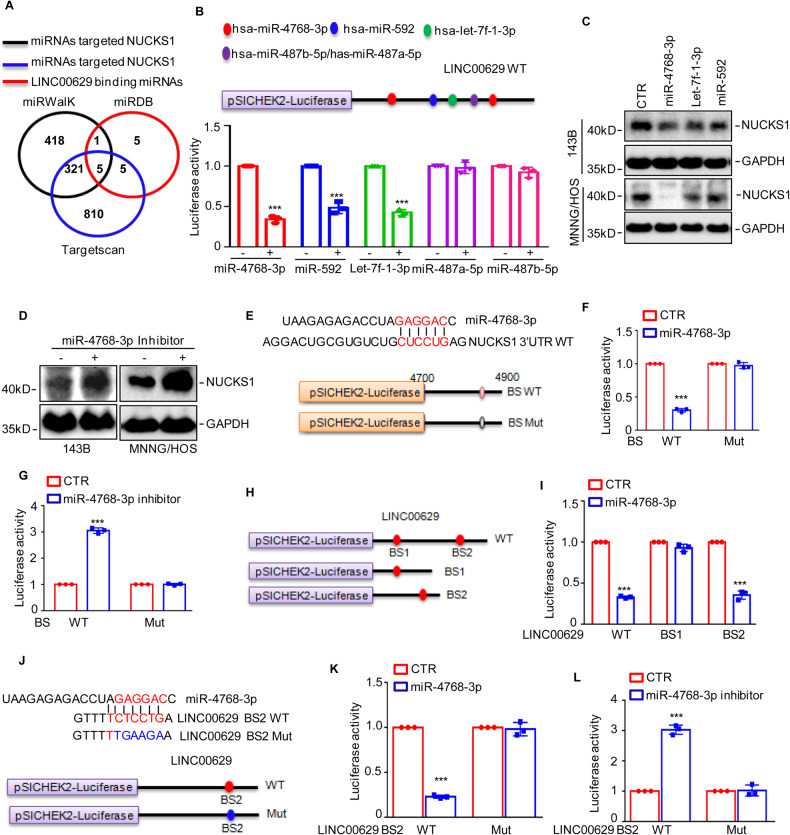
LINC00629 activates NUCKS1/ASNS axis via sponging miR-4768-3p. **A** The potential miRNAs targeted NUCKS1 were predicted from miRwalk database (black line) and Targetscan database (blue line). The potential miRNAs targeted NUCKS1 were predicted from miRDB (red line). The five overlapped miRNAs were obtained. **B** The binding site of the five miRNAs on LINC00629 were listed and the effects of miRNAs on LINC00629 were assessed. **C** The indicated miRNAs were transfected into MNNG/HOS and 143B cells. The expression of NUCKS1 was detected by western blot. **D** miR-4768-3p inhibitor were transfected into MNNG/HOS and 143B cells. The expression of NUCKS1 was detected by western blot. **E** Potential binding site of miR-4768-3p on NUCKS1 3’UTR was shown. The wild-type binding site or mutant of miR-4768-3p on NUCKS1 3’UTR were cloned into a pSICHECK2 vector. The blue indicates the mutated region. **F** The WT or Mut of NUCKS1 3′UTR was respectively transfected into MNNG/HOS cells with or without miR-4768-3p overexpression. The luciferase activity of NUCKS1 3′UTR was measured. **G** The WT and Mut of NUCKS1 3’UTR was transfected into MNNG/HOS cells with or without miR-4768-3p inhibitor treatment. The luciferase activity of NUCKS1 3’UTR was measured. **H** Schematic illustration of pSICHECK2 reporter constructs used in luciferase assays to examine the effects of miR-4768-3p on luciferase activity of LINC00629 and different truncations named BS1 and BS2. **I** the full length of LINC00629 and the different truncations were transfected into MNNG/HOS cells with or without miR-4768-3p overexpression. The luciferase activity was measured. **J** The BS2 wild type and mutant were constructed into pSICHEK2 vector. **K**, **L** BS2 WT and Mut were transfected into MNNG/HOS cells with or without miR-4768-3p or miR-4768-3p inhibitor. The luciferase activity was measured. Data in **B**, **F**, **G**, **I**, **K** and **L** were analyzed by Student’s *t* test, *p < 0.05, **p < 0.01, ***p < 0.001.

A previous study indicated that LINC00629 upregulated AQP4 expression by competitively binding to miR-196b-5p [33]. We thus wanted to determine whether LINC00629 elevated NUCKS1 expression by competitively binding to miRNAs. To this end, we used the miRWalk and TargetScan databases to predict the miRNAs that targeted the 3’UTR of NUCKS1. We also observed the potential miRNAs that bound to LINC00629. These miRNAs observed from different databases were combined in a Venn Diagram, and the overlapping miRNAs were identified (Fig. 8A). The overlapping miRNAs were listed and then separately transfected into MNNG/HOS cells together with LINC00629 expressed in the pSICHECK2 vector. The luciferase activities were measured. The results indicated that miR-4768-3p, miR-592, and let-7f-1-3p, but not miR-487b-5p and miR-487a-5p, significantly downregulated the luciferase activities of LINC00629 (Fig. 8B). These data suggested that miR-4768-3p, miR-592 and let-7f-1-3p may bind to LINC00629. Subsequently, we transfected miR-4768-3p, miR-592, and let-7f-1-3p into osteosarcoma cells, and the expression of NUCKS1 was detected by Western Blotting. As illustrated in Fig. 8C, we observed that miR-4768-3p markedly reduced NUCKS1 expression in MNNG/HOS and 143B cells. However, inhibition of miR-4768-3p increased NUCKS1 protein levels (Fig. 8D). To further verify this hypothesis, the wild-type or mutant 3ʹUTR of NUCKS1 containing the putative miR-4768-3p binding site was cloned into a pSICHECK2 vector (Fig. 8E). Then, these plasmids were transfected into MNNG/HOS cells with or without miR-4768-3p, and we found that the luciferase activity of the wild-type 3ʹUTR of NUCKS1 was decreased in miR-4768-3p-overexpressing cells. However, the decrease in luciferase activity disappeared when the binding site was mutated (Fig. 8F). Similarly, the miR-4768 inhibitor elevated the luciferase activity of the wild-type NUCKS1 3’UTR and had no effect on the mutant (Fig. 8G). These data indicated that miR-4768-3p targeted NUCKS1 and suppressed its expression in osteosarcoma cells.

Furthermore, to identify the positive binding site of miR-4768 on LINC00629 and confirm their relationship, we inserted full-length LINC00629 and two truncations into the pSICHECK2 vector, which were named WT, BS1, and BS2 (Fig. 8H). As shown in Fig. 8I, we found that miR-4768-3p suppressed the luciferase activities of LINC00629 WT and BS2 and had no effect on the activity of BS1, indicating that BS2 is a true binding site of miR-4768-3p on LINC00629. To further confirm this hypothesis, wild-type or mutant LINC00629 BS2 was cloned into a pSICHECK2 vector (Fig. 8J). Then, these plasmids were transfected into MNNG/HOS cells with or without miR-4768-3p, and we found that miR-4768-3p suppressed the luciferase activities of LINC00629 BS2 WT and had no effect on BS2 Mut (Fig. 8K). Consistently, inhibition of miR-4768-3p elevated the luciferase activities of LINC00629 BS2 WT and had no effect on BS2 Mut (Fig. 8L). Collectively, our data indicate that LINC00629 acts as a miR-4768-3p sponge and promotes NUCKS1 expression in osteosarcoma cells.

### LINC00629 activated the NUCKS1/ASNS axis and promoted asparagine synthesis by completely binding to miR-4768-3p

**Fig. 9:**
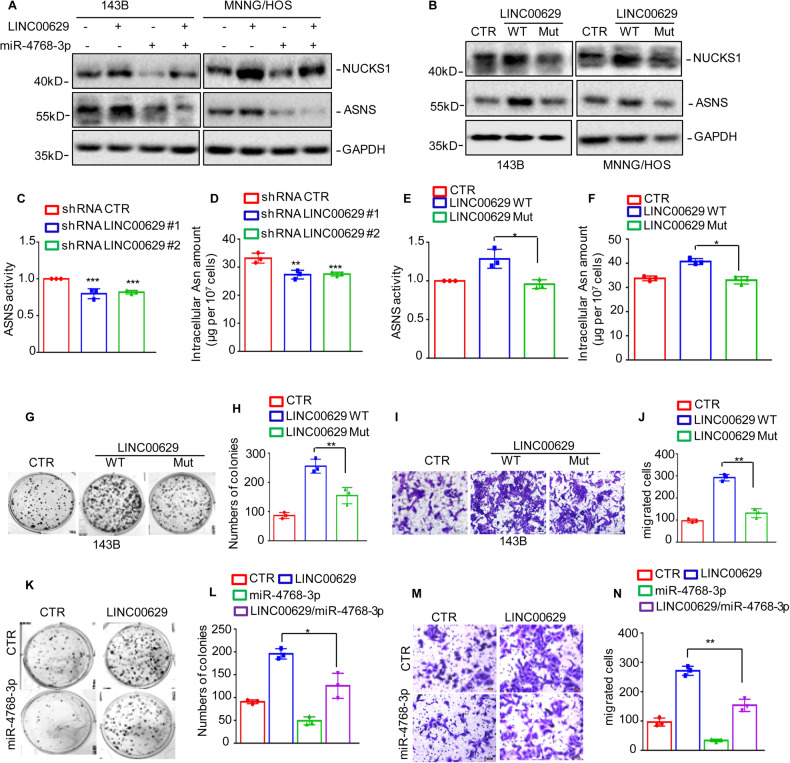
LINC00629 activated NUCKS1/ASNS axis and promoted Asparagine synthesis through completely binding to miR-4768-3p. **A** miR-4768-3p was transfected into 143B and MNNG/HOS cells with or without LINC00629 overexpression. The expression levels of NUCKS1 and ASNS were detected by western blot. **B** The wild type (WT) of LINC00629 or the mutational binding site the miR-4768-3p (Mut) were transfected into 143B and MNNG/HOS cells. The expression levels of NUCKS1 and ASNS were detected by western blot. **C**, **D** The enzyme activity of ASNS and the intracellular Asn amount were assessed in MNNG/HOS cells with or without LINC00629 knockdown. **E**, **F** The WT or Mut of LINC00629 was transfected into MNNG/HOS cells. The enzyme activity of ASNS and the intracellular Asn amount were assessed. **G**–**J** The WT or Mut of LINC00629 was transfected into MNNG/HOS cells. Cell growth and migration were assessed by colony formation and Transwell assays. **K**–**N** miR-4768-3p was transfected into MNNG/HOS cells with or without LINC00629 overexpression. Cell growth and migration were assessed by colony formation and Transwell assays. Data in **C**–**F**, **H**, **J**, **L** and **N** were analyzed by Student’s *t* test, *p < 0.05, **p < 0.01, ***p < 0.001.

To prove whether LINC00629 elevated NUCKS1 and ASNS expression by binding to miR-4768-3p, we first transfected miR-4768-3p into 143B and MNNG/HOS cells with or without LINC00629 overexpression. As shown in Fig. 9A, overexpression of miR-4768-3p abolished the increase in NUCKS1 and ASNS induced by LINC00629. To confirm this hypothesis, we then inserted full-length LINC00629 containing the wild-type binding site (WT) of miR-4768-3p or the mutational binding site (Mut) into the pCDH vector. Similarly, LINC00629 WT significantly elevated NUCKS1 and ASNS expression. However, the elevation was abolished when the binding site was mutated (Fig. 9B).

The subsequent results indicated that depletion of LINC00629 also decreased ASNS activity and Asn levels (Fig. 9C, D). However, overexpression of LINC00629 WT elevated ASNS activity and Asn levels. However, the elevation was reversed by LINC00629 Mut (Fig. 9E, F). In addition, we further investigated whether LINC00629 promoted osteosarcoma cell proliferation and migration by acting as a sponge to miR-4768-3p. Our data indicated that LINC00629 facilitated osteosarcoma cell growth and migration. However, the promotion was impaired by LINC00629 Mut or miR-4768-3p (Fig. 9G–N). Collectively, these data suggest that LINC00629 activates the NUCKS1/ASNS axis and promotes asparagine synthesis by completely binding to miR-4768-3p.

## Discussion

In this work, we found that NUCKS1 played an oncogenic role in osteosarcoma and promoted asparagine synthesis by upregulating ASNS expression. Increased ASNS or asparagine production by NUCKS1 facilitated cell growth and metastasis. In addition, we also reported that LINC00629 upregulated NUCKS1 and ASNS expression by completely binding miR-4768-3p.

NUCKS1 is a member of the high mobility group family of proteins and participates in a number of biological processes, including the DNA damage response, cell cycle control, and energy metabolism [9, 11, 34]. An increasing number of studies have revealed that NUCKS1 is increased in multiple cancers and involved in tumor development. For example, NUCKS1 was significantly upregulated in hepatocellular carcinoma and acted as a novel biomarker for the prognosis of patients with hepatocellular carcinoma [20]. NUCKS1 promotes gastric cancer cell aggressiveness by regulating autophagy [19]. In lung cancer, NUCKS1 promoted cell growth, migration, and invasion through the PI3K/Akt signaling pathway [35]. Similarly, our data uncovered the role of NUCKS1 in osteosarcoma and found that NUCKS1 facilitated osteosarcoma cell growth and metastasis.

The tumor promotion mechanism of NUCKS1 was investigated by RNA sequencing analysis, and NUCKS1 transcriptionally upregulated ASNS expression by binding its promoter. NUCKS1 is not a typical transcription factor that does not have a transcription activation domain. However, previous studies indicated that NUCKS1 could bind to the promoter of SKP2 and transcriptionally upregulate its expression [11]. NUCKS1 can also transcriptionally activate insulin receptor (IR) expression by promoting chromatin accessibility and recruiting RNAPII to the promoter of IR [9]. Therefore, it is possible that NUCKS1 elevates ASNS expression via chromatin alterations or cooperation with other transcription factors to direct transcription. The precise mechanism needs to be further investigated in our future work.

ASNS consists of 561 amino acid residues and can convert aspartate and glutamine to asparagine and glutamate in an ATP-dependent manner [22]. Several transcription factors were reported to regulate ASNS expression and contribute to ASNS upregulation in cancers. For instance, ATF4 transcription upregulates ASNS and contributes to apoptotic suppression, protein biosynthesis and mTORC1 activation [36, 37]. Zinc finger and BTB domain-containing protein 1 (ZBTB1) promotes ASNS expression by binding the promoter of ASNS, which mediates the sensitivity of leukemia cells to L-asparaginase [38]. The well-known tumor suppressor p53 was reported to downregulate ASNS expression and lead to lymphoma and colon tumor growth inhibition in vitro and in vivo [39]. Similarly, our findings reported that NUCKS1 was a new regulator of ASNS and identified ASNS as a downstream gene of NUCKS1 that promoted osteosarcoma cell growth and metastasis.

Subsequently, we found that LINC00629 elevated NUCKS1 in osteosarcoma. LINC00629 is a long, intergenic, noncoding RNA mapped to chromosome X (Xq26). LINC00629 was reported to suppress tumor progression by upregulating AQP4 and competitively binding to miR-196b-5p in gastric cancer [33]. LINC00629 was induced by apigenin and promoted cell apoptosis in oral squamous cell carcinoma [40]. Our recent work has indicated that LINC00629 plays an oncogenic role in osteosarcoma by elevating KLF4 stability [32]. Consistently, our data showed that LINC00629 acted as a miR-4768-3p sponge and upregulated NUCKS1 expression in osteosarcoma.

Taken together, our findings highlight the role of NUCKS1 in regulating asparagine metabolism and reveal that LINC00629 is an important regulator of NUCKS1 that contributes to NUCKS1 upregulation in osteosarcoma.

## Supplementary information

supplementary figure legends

Supplementary Figure 1

Supplementary Figure 2

Supplementary Table 1

Uncropped WB picture

Aj-checklist

## Data Availability

All data generated or analyzed during this study are included in this published article.
