# Supplementary material for: NUCKS1, a LINC00629-upregulated gene, facilitated osteosarcoma progression and metastasis by elevating asparagine synthesis
Source: Cell Death Dis. 2023 Aug 1;14(8):489. doi: 10.1038/s41419-023-06010-9 (PMC10393983; doi:10.1038/s41419-023-06010-9)
Supplement: Supplementary file 1 — supplementary figure legends [file 41419_2023_6010_MOESM1_ESM.docx]

Supplementary Figure 1(A-D) 10μM L-Asparaginase was added in the indicated osteosarcoma cells with or without NUCKS1 overexpression. Cell growth and migration were assessed by colony formation and Transwell assays. (E-F) The indicated143B cells (10^6^ cells per mouse) were injected intravenously into nude mice (n=5 per group). Representative images of lung were shown and the weight of lung was analyzed. Data in B, D and F were analyzed by Student’s t test, *p < 0.05, **p < 0.01, ***p < 0.001.

Supplementary Figure 2 (A) The mRNA levels of NUCKS1 were detected by qRT-PCR in MNNG/HOS and 143B cells with or without LINC00629 knockdown. (B) MNNG/HOS cells with or without LINC00629 knockdown were treated with 10 mg/ml cycloheximide (CHX) for the indicated times. The expression levels of NUCKS1 were detected by Western blot.

Supplementary Table 1 the altered genes in NUCKS1-depleted cells.
