# Supplementary figures and images for: NUCKS1, a LINC00629-upregulated gene, facilitated osteosarcoma progression and metastasis by elevating asparagine synthesis
Source: Cell Death Dis. 2023 Aug 1;14(8):489. doi: 10.1038/s41419-023-06010-9 (PMC10393983; doi:10.1038/s41419-023-06010-9)

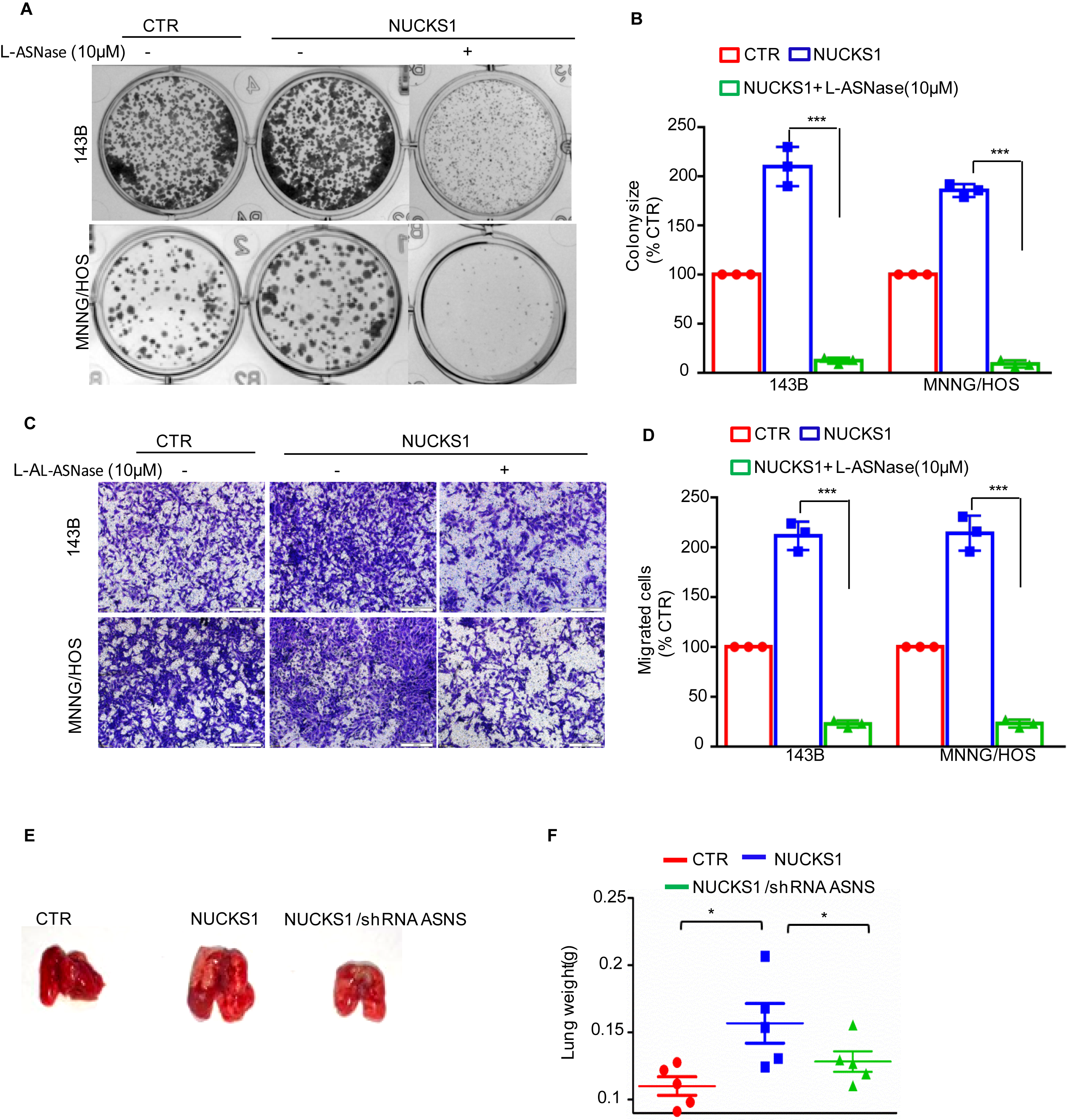

Supplement: Supplementary file 2 — Supplementary Figure 1 [file 41419_2023_6010_MOESM2_ESM.tif]

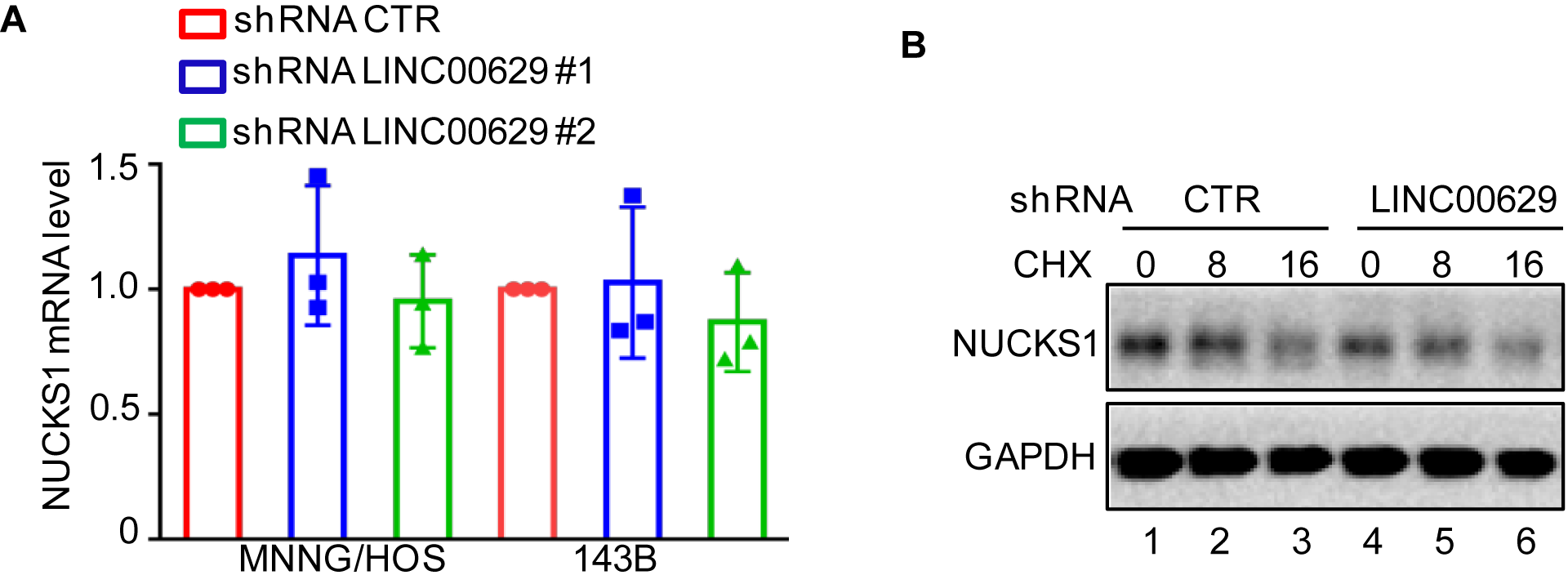

Supplement: Supplementary file 3 — Supplementary Figure 2 [file 41419_2023_6010_MOESM3_ESM.tif]
