# Supplementary material for: NUCKS1, a LINC00629-upregulated gene, facilitated osteosarcoma progression and metastasis by elevating asparagine synthesis
Source: Cell Death Dis. 2023 Aug 1;14(8):489. doi: 10.1038/s41419-023-06010-9 (PMC10393983; doi:10.1038/s41419-023-06010-9)

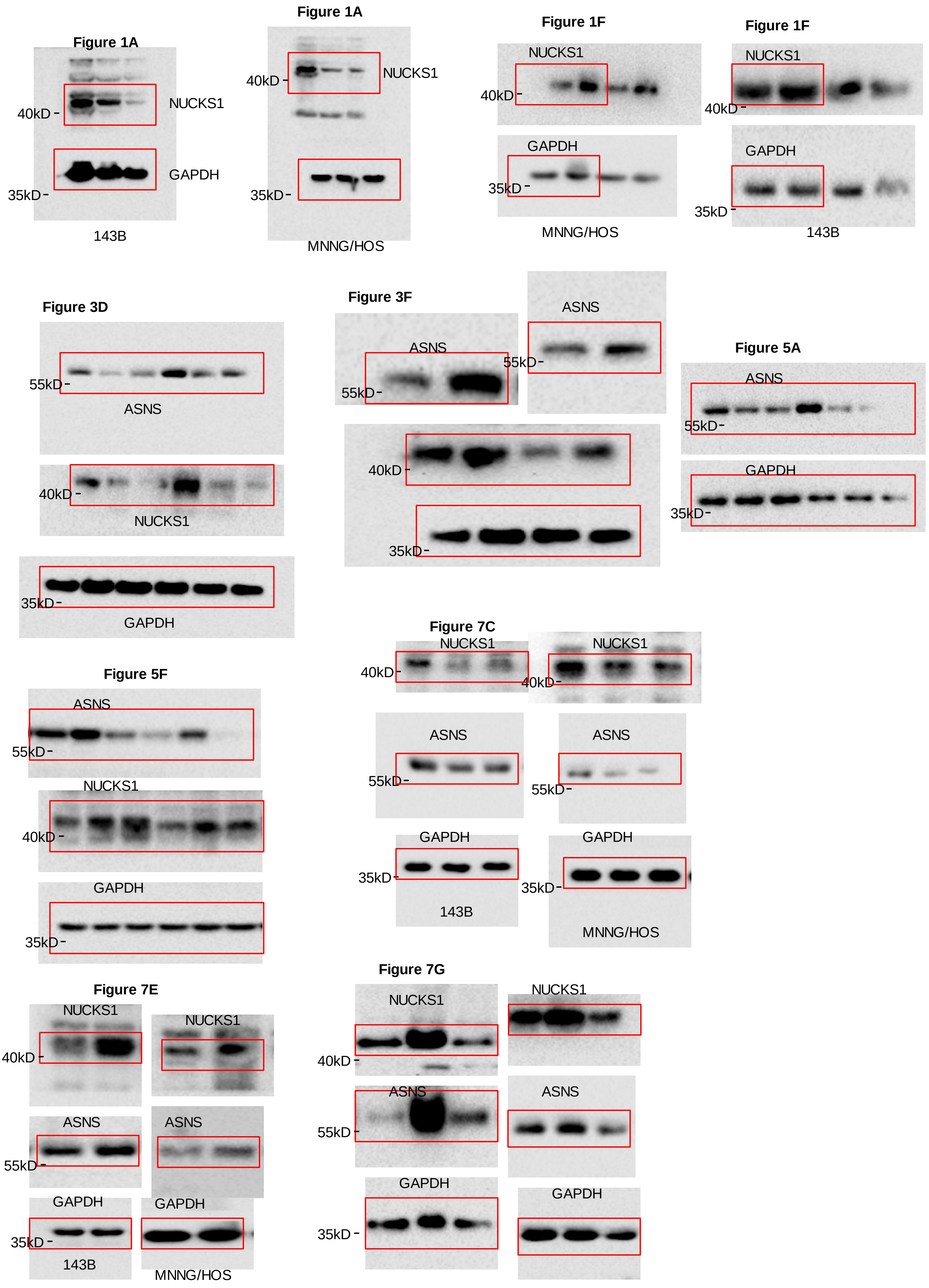
Figure 1A
NUCKS1
GAPDH
40kD
35kD
143B
Figure 1A
NUCKS1
40kD
35kD
MNNG/HOS
Figure 1F
NUCKS1
GAPDH
40kD
35kD
MNNG/HOS
Figure 1F
NUCKS1
GAPDH
40kD
35kD
143B
Figure 3D
ASNS
NUCKS1
GAPDH
55kD
40kD
35kD
Figure 3F
ASNS
ASNS
55kD
55kD
40kD
35kD
Figure 5A
ASNS
GAPDH
55kD
35kD
Figure 5F
ASNS
NUCKS1
GAPDH
55kD
40kD
35kD
Figure 7C
NUCKS1
NUCKS1
ASNS
ASNS
GAPDH
GAPDH
40kD
40kD
55kD
55kD
35kD
35kD
143B
MNNG/HOS
Figure 7E
NUCKS1
NUCKS1
ASNS
ASNS
GAPDH
GAPDH
40kD
55kD
35kD
143B
MNNG/HOS
Figure 7G
NUCKS1
NUCKS1
ASNS
ASNS
GAPDH
GAPDH
40kD
55kD
35kD

Figure 8C

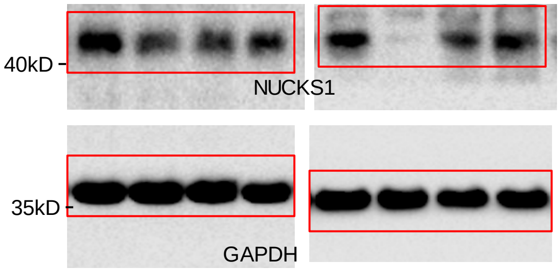

Figure 8D

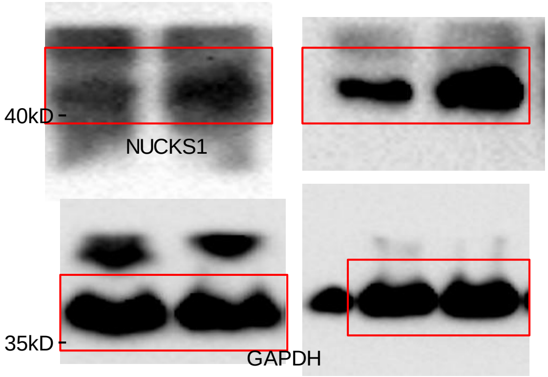

Figure 9A

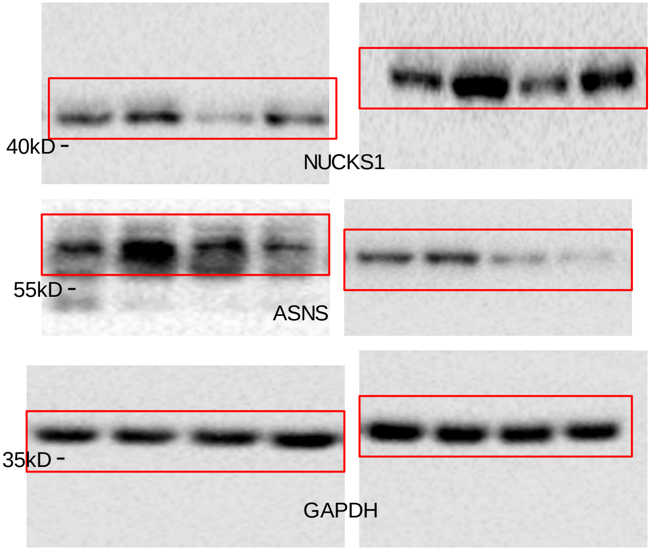

Figure 9B

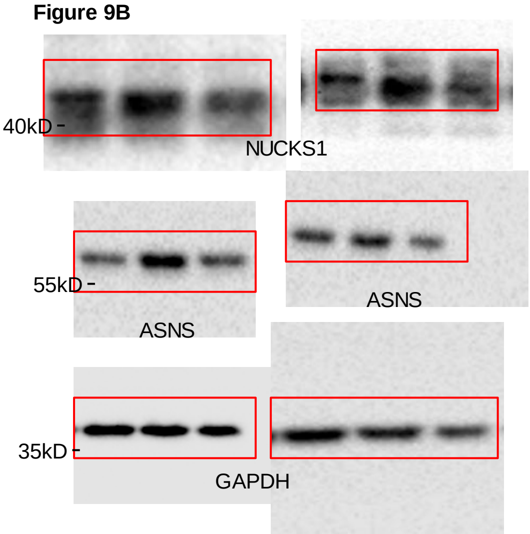

Supplementary Figure 2B

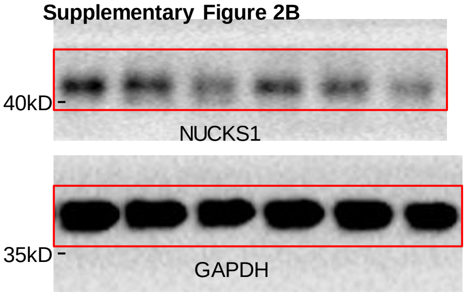

Supplement: Supplementary file 5 — Uncropped WB picture [file 41419_2023_6010_MOESM5_ESM.pdf]
